# Transition of patients with metabolic bone disease from paediatric to adult healthcare services: current situation and proposals for improvement

**DOI:** 10.1186/s13023-023-02856-6

**Published:** 2023-08-29

**Authors:** Enrique Casado, Carlos Gómez-Alonso, Guillem Pintos-Morell, Rosa Bou-Torrent, Ana Coral Barreda-Bonis, José Vicente Torregrosa, José Jesús Broseta-Monzó, Pedro Arango-Sancho, Sara Chocrón-de-Benzaquen, Yoko Olmedilla-Ishishi, Begoña Soler-López

**Affiliations:** 1 Rheumatology Department, Hospital Universitario Parc Taulí, Sabadell, Barcelona Spain; 2 Bone and Mineral Metabolism Clinical Management Unit, Hospital Universitario Central de Asturias, Oviedo, Spain; 3 Hereditary Metabolic Diseases, Hospital Universitario Vall d’Hebron, Barcelona, Spain; 4 Paediatric Rheumatology Unit, Hospital Sant Joan de Déu, Barcelona, Spain; 5 Paediatric Endocrinology Department, Hospital Universitario la Paz, Madrid, Spain; 6 Department of Nephrology and Renal Transplant, Hospital Clínic de Barcelona, Barcelona, Spain; 7 Department of Paediatric Nephrology, Hospital Sant Joan de Déu, Barcelona, Spain; 8 Department of Paediatric Nephrology, Hospital Universitario Vall d’Hebron, Barcelona, Spain; 9 Medical Department, Kyowa Kirin Farmacéutica, S.L., Madrid, Spain; 10 Medical Department, E-C-BIO, S.L., c/Rosa de Lima, 1, Edificio ALBA, Office 016, 28230 Las Rozas, Madrid Spain

**Keywords:** Transitional care, Paediatric, Metabolic bone disease, X-linked hypophosphataemia (XLH)

## Abstract

**Background:**

There are currently no models for the transition of patients with metabolic bone diseases (MBDs) from paediatric to adult care. The aim of this project was to analyse information on the experience of physicians in the transition of these patients in Spain, and to draw up consensus recommendations with the specialists involved in their treatment and follow-up.

**Methods:**

The project was carried out by a group of experts in MBDs and included a systematic review of the literature for the identification of critical points in the transition process. This was used to develop a questionnaire with a total of 48 questions that would determine the degree of consensus on: (a) the rationale for a transition programme and the optimal time for the patient to start the transition process; (b) transition models and plans; (c) the information that should be specified in the transition plan; and (d) the documentation to be created and the training required. Recommendations and a practical algorithm were developed using the findings. The project was endorsed by eight scientific societies.

**Results:**

A total of 86 physicians from 53 Spanish hospitals participated. Consensus was reached on 45 of the 48 statements. There was no agreement that the age of 12 years was an appropriate and feasible point at which to initiate the transition in patients with MBD, nor that a gradual transition model could reasonably be implemented in their own hospital. According to the participants, the main barriers for successful transition in Spain today are lack of resources and lack of coordination between paediatric and adult units.

**Conclusions:**

The TEAM Project gives an overview of the transition of paediatric MBD patients to adult care in Spain and provides practical recommendations for its implementation.

**Supplementary Information:**

The online version contains supplementary material available at 10.1186/s13023-023-02856-6.

## Background

The process of transition from paediatric to adult care has acquired considerable interest in recent years in patients with childhood-onset chronic diseases [1]. The lack of coordination between specialists and paediatric and adult services, which is well documented in the literature, and the lack of proper training in the transition process, make clinical management of adolescent patients more difficult, and may have a negative impact on their disease [2–9].

Consultation with the patient during the transition period requires designating the necessary time to foster mutual respect, promote independence in the management of their disease, strengthen the doctor-patient relationship, and thus treat not only the patient’s underlying condition, but also important health-related aspects beyond the disease itself [4, 8, 10–12].

Process planning coordinated between paediatric and adult specialists—widespread in diseases such as diabetes or arthritis, but in its infancy in some metabolic bone diseases (MBDs) [13]—has been shown improve quality of care, although further studies are still needed to assess the quality and effectiveness of transition programmes [14–25]. There is therefore a broad consensus that this planning should be considered in patients with any chronic disease [1]. In fact, the need to develop transition programmes for adolescents with chronic diseases to prevent deterioration in their health is defined in different regulations in United Kingdom, Canada and the United States [26–36].

Nevertheless, despite the fact that physicians agree on the need for transition programmes [3], they are rarely applied in clinical practice [37, 38], and fewer than 15% of adolescents who could be included actually participate in these schemes [39].

Although different scientific societies have developed consensus documents, guidelines and recommendations in recent years to guide the rollout of transition units, there are still not defined transition models for patients with MBDs, from the most common entities such as secondary osteoporosis or osteogenesis imperfecta, to the rarest such as acquired and congenital hypophosphataemic rickets or X-linked hypophosphataemia (XLH).

In the specific case of XLH, because there is no clear consensus on how to make the transition from paediatric to adult-oriented care, many questions remain to be answered in future guidelines or recommendations. One of these questions concerns the right age to begin the transition process from paediatric to adult care. In this respect, Dahir et al. suggest that preparation for transition can begin when patients with XLH are around 12 years old, and continue until transfer to care by adult clinicians at approximately 18–26 years [13]. Consensus, furthermore, needs to be reached on how this transition should be approached and managed in a multidisciplinary manner by the medical/care team, due to the multisystemic nature of XLH [40, 41]. Some recently published European consensus documents and guidelines on the management of patients with XLH have highlighted the need to implement and anticipate a multidisciplinary approach to transition [42–44]. In the Belgian setting, similar to Spain in that the treatment of MBD does not fall under the umbrella of a specific medical specialty recognized as such, it has been suggested that several specialties should be involved, either in the routine follow-up or as possible interdepartmental consultation. These should be both paediatric and adult specialties, including endocrinologists, nephrologists, rheumatologists, orthopaedics specialists, neurosurgeons, radiologists, geneticists, specialists in physical medicine and rehabilitation, dentists, orthodontists and maxillofacial surgeons, and even urologists, otorhinolaryngologists and ophthalmologists [42]. However, it should be noted that more barriers or difficulties than usual may be encountered in managing the transition in patients with XLH, due to a lack of knowledge about the disease among many of the specialists potentially involved in it.

The objective of the TEAM Project (*Transición a la Edad Adulta de pacientes con enfermedades Metabólicas óseas*, Transition to adult care of patients with metabolic bone diseases) was to seek the views and experience of physicians regarding the existing transition units in Spain for patients with MBD or similar disease processes, in order to identify and define areas for improvement, and to develop practical recommendations for patient management in the transition to adult care that are presented in this document.

## Methods

### Study design and ethical standards

A committee of nine experts in MBD in children (paediatric rheumatology, nephrology and endocrinology) and adult specialists (rheumatology and internal medicine) was selected in base of their expertise and scientific background in bone metabolism. The objectives of the committee were:

- To analyse the information from a systematic literature review on transition units, and, more specifically, in patients with MBD. For this review, a systematic review protocol was designed to identify consensus-type articles, guidelines, and systematic reviews in Spanish and English, following PRISMA guidelines [45]. The websites of various international institutions with information on transition programmes for children and adolescents with different diseases were also consulted. The experts committee discussed on networking the systematic review documents to be discussed in next steps.
- To define the indicators that would be submitted to a Delphi consensus process. One face-to-face meeting was completed to select 48 questions grouped into five survey sections (Additional file 1). The survey was checked out by the experts and their local teams. After one online meeting, the final survey was obtained. The survey was distributed to medical specialists from eight scientific societies on a website that met the CHERRIES quality standards for electronic questionnaires [46].
- To develop a series of recommendations for the management of patients with MBD during the transition period, based on the results of the consensus. Two online experts’ meetings were needed for the recommendation’s consensus.

The action in patients with XLH was specifically asked about, since this is considered to be the MBD entity with the greatest effect on different organs and systems, and the one that requires more intense clinical follow-up at the transition age.

The TEAM Project was authorized by the Clinical Research Ethics Committee of the Hospital Universitario Parc Taulí de Sabadell (Spain) on 28-07-2020 (Reference 2020/695). Data were collected from 1 June to 10 December 2021.

### Participants

The questionnaire was completed by physicians involved in the management of patients with MBD during the period of transition to adult care. Physicians were members of one of the scientific societies that endorsed the project: AECOM (Spanish Association for the Study of Inborn Errors of Metabolism), AENP (Spanish Association of Paediatric Nephrology), SEEN (Spanish Society of Endocrinology and Nutrition), SEEP (Spanish Society of Paediatric Endocrinology), SEIOMM (Spanish Society of Bone and Mineral Metabolism Research), SEMI (Spanish Society of Internal Medicine), SEN (Spanish Society of Nephrology) and SERPE (Spanish Society of Paediatric Rheumatology).

### Statistical methods

The questionnaire included four types of questions: multiple choice, quantitative response, free text, and questions to indicate the degree of agreement on a scale of 1–10 points, where the value 1 represents “no agreement” and the value 10 represents “total agreement” with the statement of the question. The results of the multiple-choice questions are shown as the number and proportion of participants who chose each answer. Free text questions were coded into categories and described in the same way as above. Quantitative questions are shown as number of responses, mean value, and 95% confidence interval (CI). The results of the questions about the degree of agreement with a statement were analysed quantitatively, expressing the mean score of the degree of agreement, and the proportion of participants who responded to each category from 1 to 10 was calculated. Consensus was considered to have been reached on each statement when at least 70% of the participants rated their agreement at ≥ 7 points. IBM-SPSS 27.0 software was used for statistical analysis.

## Results

A total of 86 physicians from 53 hospitals located in 23 Spanish provinces participated, with representation from most autonomous regions of the country.

Almost all the respondents (97.7%) had experience in managing patients with MBD, with 52.3% being part of a transition unit, clinic or programme. The mean age of participants was 47 years and 55.8% were women. Half were paediatricians (52.3%) and 40.7% were adult physicians working in public hospitals (87.2%). The most common specialty of the physicians surveyed was nephrology (43%), followed by endocrinology (20.9%), rheumatology (16.3%), paediatric medicine (12.8%) and internal medicine (7%). Doctors had an average of 18 years of experience in their specialty, and 23.1% (95% CI 15.1–31) of patients in their clinics had been diagnosed with MBD.

Consensus was reached in a single round of the Delphi questionnaire. Table 1 describes the results of the consensus questions on the implementation and timing of the transition. Consensus was reached on all but two statements: “It is appropriate to start the transition programme at 12 years of age in patients with MBD", which obtained a mean score of 5.42 (95% CI 4.8–6), with agreement reached in 36% of physicians; and the statement “It is feasible to start the transition programme at 12 years of age in patients with MBD”, with a mean score of 5.1 (95% CI 4.5–5.7), and agreement was reached in 29.4% of participants.

**Table 1:** Results of the consensus on transition indicators and timing

	N	%	N	Mean	95% CI	Proportion of physicians with agreement ≥ 7 points (%)
Understands what a transition unit, clinic or programme is						
No	2	2.3				
Yes	84	97.7				
A care transition programme needs to be created for children with metabolic bone diseases who transfer from paediatric to adult services			86	9.1	8.9–9.4	97.7
It is feasible to establish a transition programme for children with metabolic bone diseases in your setting			86	7.6	7.1–8	75.6
Age at which you think it is best to start the transition programme in children with metabolic bone diseases			86	14.7	14.4–15.1	
It is appropriate to start the transition programme at 12 years old in patients with metabolic bone diseases			86	5.4	4.8–6	36*
It is feasible to start the transition programme at 12 years old in patients with metabolic bone diseases			85	5.1	4.5–5.7	29.4*
Age at which you think it is best to start the transition programme for children with X-linked hypophosphataemia (XLH)			85	14.6	14.3–15	
In patients with XLH, which medical specialty should be the primary case manager in paediatrics?						
I don't know	1	1.2				
It depends on the specialist's experience	3	3.5				
Nephrology	39	45.3				
Endocrinology	14	16.3				
Metabolic diseases	3	3.5				
Nephrology–endocrinology	9	10.5				
Nephrology-bone metabolism unit	1	1.2				
Paediatric medicine	7	8.1				
Rheumatology	7	8.1				
Rheumatology–endocrinology	2	2.3				
Internal medicine	0	0				
Bone metabolism	0	0				
Nephrology–rheumatology	0	0				
Nephrology–endocrinology–rheumatology	0	0				
Multidisciplinary unit in charge of rare diseases	0	0				
In XLH patients, which medical specialty should be the primary case manager in adults?						
I don't know	1	1.2				
It depends on the specialist's experience	4	4.7				
Nephrology	25	29.1				
Endocrinology	19	22.1				
Metabolic diseases	1	1.2				
Nephrology–endocrinology	4	4.7				
Nephrology-bone metabolism unit	0	0				
Paediatric medicine	0	0				
Rheumatology	14	16.3				
Rheumatology–endocrinology	1	1.2				
Internal medicine	5	5.8				
Bone metabolism	5	5.8				
Nephrology–rheumatology	4	4.7				
Nephrology–endocrinology–rheumatology	2	2.3				
Multidisciplinary unit in charge of rare diseases	1	1.2				
Validated scales must be used to evaluate whether the adolescent is ready to start follow-up in adult specialties			86	7.8	7.3–8.2	73.3

*The indicator did not reach consensus

Table 2 shows the consensus results on the indicators for the model and transition plan, where consensus was reached on all but one question: “Is the transition model you selected as your preferred model feasible in your setting?” A mean score of 7.1 (95% CI 6.6–7.6) was obtained and consensus was reached in 68.6% of participants.

**Table 2:** Results of the consensus on model and transition plan indicators

	N	%	N	Mean	95% CI	Proportion of physicians with agreement ≥ 7 points (%)
There is a transition model or plan in your hospital						
No	56	65.1				
Yes	30	34.9				
Preferred transition model in the care of patients with metabolic bone diseases						
Direct and complete transfer to the adult specialist when patient turns 18	3	3.5				
Gradual transfer to the adult specialist, with multidisciplinary paediatric-adult consultation during the transition	76	88.4				
The patient is seen both in a paediatric clinic and an adult clinic, in different specialties	2	2.3				
No transition, the patient remains with the paediatric specialist in the adult phase as well	1	1.2				
Other type of transition model	2	2.3				
None of the options	2	2.3				
Is the transition model you selected as your preferred model feasible in your setting?			86	7.1	6.6–7.6	68.6*
There should be a transitional care unit/programme manager or case manager			86	8.8	8.5–9.1	95.3
It is feasible in your setting to appoint a transitional care unit/programme manager or case manager			86	7.7	7.2–8.1	74.4
Multidisciplinary working groups should be created for the management of adolescents with metabolic diseases			86	9.1	8.8–9.4	95.3
It is feasible in your setting to create multidisciplinary working groups for the management of adolescents with metabolic diseases			86	7.7	7.3–8.1	81.4

*The indicator did not reach consensus

Consensus was reached on all statements regarding the information needed in the transition program (Table 3).

**Table 3:** Results of the consensus in relation to the information needed in the transition programme

	N	Mean	95% CI	Proportion of physicians with agreement ≥ 7 points (%)
Parents should be informed about the transition programme at the time the metabolic bone disease is diagnosed	86	7.5	6.9–8.1	73.3
Minimum information that should be provided to parents and the child during the transition programme:				
Letter of information for parents	86	8.2	7.7–8.7	83.7
Letter of information for the adolescent	86	8.1	7.6–8.6	81.4
Names and contact details of paediatric and adult specialists involved in patient care and contact details for nurse, social worker, and case manager	86	8.8	8.5–9.2	91.9
Rough outline of visits and scheduled tests until the transition to adult care is complete	86	8.4	8–8.8	87.2
Epidemiology of the disease, prevalence, incidence	86	7.8	7.3–8.2	76.7
Diagnosis of the disease	86	8.7	8.3–9.1	90.7
Prognosis of the disease, possible complications	86	8.8	8.5–9.1	93
Frequency of disease control tests differentiating childhood, adolescence and adulthood	86	8.3	7.9–8.6	88.4
Differences in treatment in childhood, adolescence and adulthood	86	8.3	7.9–8.6	86
General health issues, diet, working life, smoking, alcohol, substances, fertility, contraception	86	9	8.7–9.3	94.2
Patient associations and medical societies for information and advice	86	8.8	8.5–9.1	93
Passport or card with information for management of the adolescent in case of emergency	86	8.1	7.7–8.5	83.7
Specific indicators of adequate metabolic disease control need to be identified	86	8.8	8.5–9.1	95.3
Planning for the time when the adolescent comes to the clinic alone, without their parents, is necessary	86	8.2	7.8–8.6	84.9
The adolescent’s degree of knowledge of the disease and its treatments should be assessed	86	8.9	8.6–9.2	95.3
The adolescent's satisfaction with the transition programme should be assessed	86	8.8	8.4–9.1	91.9
The role of the nurse in providing parents and children with information about the transition is important	86	8.7	8.4–9.1	93
The collaboration of the social worker in providing parents and children with information about the transition is important	86	7.7	7.2–8.1	74.4

With regard to the statements on the documents needed and training on transition, consensus was reached on all the questions posed (Table 4).

**Table 4:** Consensus results on required documents and transition training

	N	Mean	95% CI	Proportion of physicians with agreement ≥ 7 points (%)
What data you think is essential in the transition report to another specialist in the care of the patient with metabolic bone disease?				
Demographic data	86	8.6	8.2–9.1	89.5
Family history of disease	86	9.4	9.1–9.7	97.7
Personal history of disease	86	9.5	9.3–9.8	97.7
Date of diagnosis of metabolic bone disease	86	9.5	9.2–9.8	95.3
History of previous treatments and efficacy obtained	86	9.6	9.3–9.8	96.5
Complications of the disease and its treatments	86	9.6	9.4–9.8	98.8
Current treatments	86	9.7	9.4–9.9	98.8
Indicators for the control of metabolic bone disease	86	9.5	9.2–9.7	98.8
Essential data to consider in the event of an emergency	86	9.4	9.1–9.7	96.5
Templates for transition documents may be useful	86	8.8	8.5–9.2	94.2
Paediatricians and specialists need to be trained in the transition to adult care programme	86	9	8.7–9.3	95.3
It is important to have accreditation in training in transition programmes	86	7.6	7.2–8.1	79.1

## Discussion

### Rationale for the transition programme and start time

Today, the need for successful healthcare transition is recognized in most chronic diseases, including MBDs. There is evidence that the process of transition to adult care in children/adolescents with chronic diseases is associated with a deterioration in their health [2, 4–7, 47, 48]. Furthermore, if the disease is diagnosed during adolescence, it will have a great physical and psychological impact on the adolescent [49]. Several surveys of adolescents with different chronic diseases and their parents highlight the need for interventions that minimize the risk of deteriorating the health status of children when they access adult services [37, 47, 50, 51]. Likewise, patients with bone diseases are monitored by different paediatric and adult specialists, depending on the disease and/or hospital, further complicating successful transition.

As described in other consensus documents [52, 53], there is also agreement among the physicians surveyed on the need for a transition programme, since the majority (97.7%) believe that they understand what a transition unit or programme is, and the same proportion consider it necessary to create a care transition programme for children with MBD. However, the percentage of agreement decrease to 75.6% when participants are asked whether it is feasible to establish a transition programme in children with MBD in their setting (Table 1).

One of the most controversial questions surrounding the transition process was the age at which it should begin. The answer is difficult, since it is a dynamic process that begins in early adolescence and ends when the patient is fully integrated into an adult unit. Most transition guidelines suggest that the process should begin when the patient is 13 or at most 14 years old [54], so that they can familiarize themselves with the adult unit prior to transfer and improve their readiness for it. In the consensus paper by Pérez-Lopez et al. in patients with inborn errors of metabolism, the authors concluded that the transition should be made at the age of 16–18 years, when the disease is in a stable phase, while taking into account the patient's level of development [55].

Although the American scientific literature considers 12 years as the recommended age for transition [56], the physicians surveyed considered that this age was neither appropriate nor feasible in Spain. For them, the most appropriate average age for initiating the transition of patients with MBD is 14.7 years, similar to that suggested for patients with XLH (14.6 years). We believe that the age of initiation should be flexible and appropriate to adolescent’s level of development and dependence. In this regard, it has been observed that doctors often consider that the patient is ready to be seen independently at an earlier age than the parents believe; therefore, a consensual decision should be sought, and the adolescent should be evaluated to determine if they are ready for transfer [9, 57–59].

Sometimes, a dependency relationship is created between the patient and parents and the paediatrician, which undermines the autonomy that the patient should be acquiring. This is because both the patient and their family perceive care in the adult unit to be of lower quality and tend to distrust it [60].

We found no references to the age of transition associated with the adolescent's gender. Physical maturation is reached 2–3 years earlier in females, and may also be accompanied by greater psychological maturity that could make transition at a younger age more viable than in males.

There was agreement on the need to use validated scales to evaluate whether the adolescent is ready to start their follow-up in adult specialties, both in the literature [61, 62] and among the physicians consulted in the survey (73.3%). However, there are few scales available to measure this variable, so the psychologist support should be desirable at this point.

#### Recommendations

**Fig. 1:**
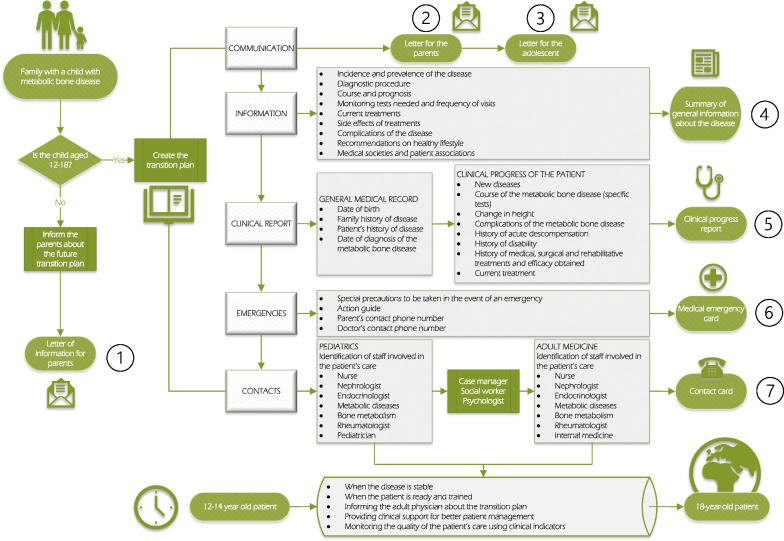
Algorithm for clinical transition of patients with metabolic bone disease from paediatric to adult care

- Parents and patients should be informed about the start of the transition process at age 12, and the process should begin at the age of 14 (Fig. 1).
- The adolescent and their family should be assessed to determine whether they are ready to begin the transition.
- Transition should begin when the patient’s metabolic bone disease is in a stable phase.

### Transition model and transition plan

The survey found that 65% of respondents did not have a transition model or plan at their facility (Table 2), although 97.7% believed it necessary (Table 1).

**Fig. 2:**
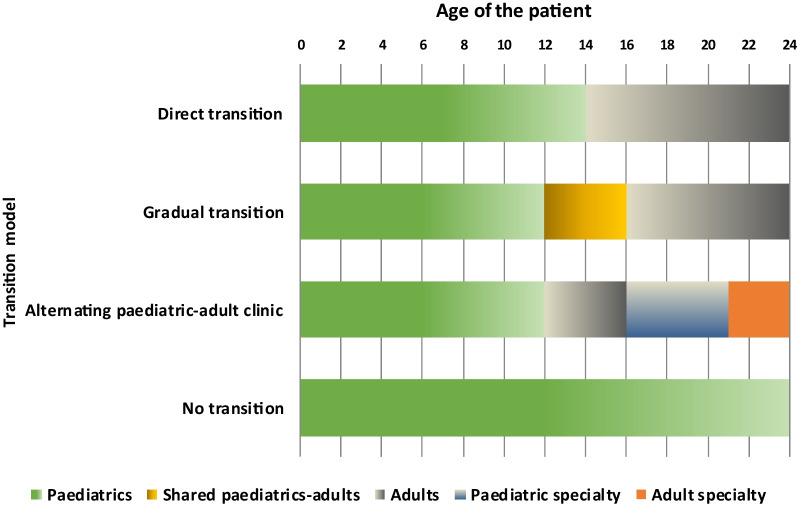
Clinical models for transition of patients from paediatric to adult care

Different transition models have been described, depending on whether the patient transfer is done directly (paediatric and adult medicine function independently and the patient is transferred with a medical report), gradually (there is a shared period between paediatric and adult medicine, which can involve joint or overlapping visits between paediatrics and the adult service), or models in which the same paediatrician manages the patient continuously throughout their paediatric and adult care. In all models, the possibility of interaction and collaboration between services is maintained once the transfer is complete (Fig. 2) [9, 63]. Most participating physicians (88.4%) preferred the transition model that consists of a gradual transfer to the adult specialist, with multidisciplinary paediatric-adult consultation during the transition. However, when asked if this model would be feasible in their hospital, the average agreement score was 7.1 points (95% CI 6.6–7.6), and only 68.6% of doctors rated their agreement above 7 points; thus, there was considered to be no agreement on this statement.

In the systematic review of the different transition models, the results were inconclusive on the differential benefit between them [1], due to various limitations: it was based on four small studies, with a limited number of clinical disorders, and follow-up of less than 12 months; moreover, the studies did not report on the specific clinical outcome resulting from use of the model in the disorder. Therefore, one model was not proven to be better than another.

Although there was agreement on the question of whether there should be a transition manager, with 95.3% of the participants rating their agreement above 7 points, a smaller proportion of doctors (74.4%) believed that it would be feasible to appoint such a person in their setting (Table 2). The existence of a contact person has been associated with higher transition success rates in patients with chronic diseases [20].

Ideally, transition units should be made up of a multidisciplinary group of healthcare professionals who coordinate and accompany the adolescent's transition to adult care. When participating physicians were asked whether multidisciplinary working groups should be created for the management of adolescents with metabolic diseases, there was consensus on the statement (mean agreement score of 9.1), with 95.3% of physicians scoring their agreement above 7 points. However, the proportion of physicians who believe it is feasible to create such groups in their setting was smaller (81.4%).

**Fig. 3:**
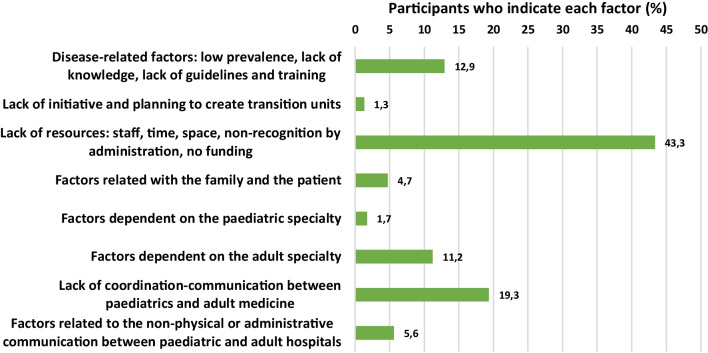
Main barriers or difficulties for the establishment of a transition programme for children with metabolic bone diseases in respondents’ setting

Therefore, there is a perceived need to set up multidisciplinary teams and appoint transition programme managers or case managers to facilitate the process, even though 8 out of 10 physicians feel it is not feasible in their setting. One of the reasons could be that specialists may be practising in different hospitals that may not share the patient's electronic record, complicating coordination between specialists, but respondents felt that the issue was mainly a lack of economic and human resources (Fig. 3).

Although the current literature on transition for chronic diseases is quite extensive in general, so far only a few hospitals have created specific programmes to ensure that transitional care for adolescents and young adults is offered in a coordinated, formalized, and standardized manner [54, 64–66]. An MBD team is defined as a core multidisciplinary group, consisting of a paediatrician/adult physician, specialized nurse and/or case manager, metabolic dietician, and a physical and rehabilitative medicine specialist, that has access to other specialists such as psychologists and social workers. Each of these various members must contribute towards providing complete information to the patient and their family, facilitated by close contact with the specialist nurse together with a detailed and comprehensive clinical report for the new team.

The ideal situation would be to ensure that, during the transition, all young people receive uninterrupted, comprehensive and accessible care within their community, so that their health does not deteriorate during this period.

#### Recommendations

- The most suitable transition model for monitoring the patient's disease should be studied and confirmed to be feasible in the particular setting.
- A case manager should be assigned to coordinate the different appointments that the patient must attend during their follow-up.
- The creation of multidisciplinary teams consisting of specialists relevant to the patient's disease is recommended. In the case of patients with MBD, this could be paediatricians and adult nephrologists, endocrinologists, rheumatologists, internal medicine and rehabilitation specialists in bone metabolism, specialized nurses and/or case managers, metabolic dieticians, physical and rehabilitative medicine specialists, psychologists and social workers.

### Information that should be specified in the transition programme

A patient who is informed about their disease and about their clinical follow-up and therapeutic alternatives will have greater decision-making capacity and will be more willing to comply with treatment. Patients want to know more about their disease, its symptoms, treatments and prognosis, but also about what other people in their situation do and feel, and they use any means at their disposal to achieve this, including information found on the internet, which can often create greater confusion.

With the right information, patients and families acquire training, empower themselves, and take full responsibility for the disease. To this end, an appropriate, gradually implemented transition programme is critical, and both families and the patient should be aware of it from the time of diagnosis (Fig. 1, point 1). The consensus on this point was 73.3% (Table 3), but this may be due to the fact that the diagnosis of some genetic diseases is made very early in life, and both the paediatrician and the family go through a difficult time, the professional in communicating information, and the family in coming to terms with a chronic disease diagnosis that may have a course and prognosis that are not always predictable. In this context, it is not surprising that the transition programme may be pushed into the background, awaiting a more stable situation. Patients should be informed and accompanied step-by-step as they gradually move from paediatric care in which physicians and caregivers are responsible for the patient in all aspects ranging from making medical appointments to administering medication, to adult care, in which the patient is aware of all recommendations, precautions and treatments needed to control their disease and avoid preventable complications and deterioration. These factors have been examined in a small study carried out in relatives and patients with rheumatic diseases [67].

Accordingly, in the initial phase of diagnosis and planning, both parents and patient should be informed (Fig. 1, point 1). The next step is to move on to a phase of training and further evaluation of the knowledge acquired for self-management (Fig. 1, points 2 and 3).

In this second phase, we recommend providing material, either written or digital, for both the parents and the adolescent (Fig. 1, point 4). Thus, the survey (Table 3) found 83.7% consensus on the need for a written report for the parents, and 81.4% for the adolescent, and a greater consensus (91.9%) that the report should contain information on names and contact details of the nurse, social worker, case manager and patient associations (Fig. 1, points 4 and 7). There was also consensus that the report should specify the visit and test schedule, the distinctive characteristics of treatment in adults (Fig. 1, point 5), information on the epidemiology, diagnosis and prognosis of the disease, and healthy lifestyle recommendations (Fig. 1, point 4). There was 83.7% consensus on the need for a personalized card for possible emergencies (Fig. 1, point 6), and 95.3% consensus on the importance of identifying specific indicators of disease control and treatment adherence.

The survey confirmed agreement on the need to plan adolescent consultations without parents (84.9%). Likewise, the ability to understand the disease and its treatments should be assessed (95.3%), as well as the satisfaction of the adolescent (91.9%), who should be encouraged to become an expert patient, well acquainted with his/her disease and of any new developments that may arise, and one who actively participates in decision-making. The adolescent’s satisfaction should be assessed periodically (at least annually) with validated scales [68], and it would also be desirable to assess the degree of training of the patient and their family in relation to disease management. The role of nursing (93%) and social work (74.4%) was highly valued for the success of the transition programme.

#### Recommendations

- It is recommended that a written transition plan be created, facilitating communication between the patient, parents and case manager with the specialist during the transition period and throughout the patient's follow-up.
- It is recommended that documents be created for the patient and their parents or guardians that contain information specific to the patient’s disease, as well as contact details for those responsible for the patient’s care during the transition.
- Templates should be created for documents such as the clinical report for transfer from paediatrics to adult specialists and a guide for action in case of emergencies and acute decompensation.

### Transition plan documents and expert training

To make the clinical transition to adult care, there was consensus among the respondents that a unifying document should be prepared that includes the materials that will be given to parents and patients, the main milestones in the history of the patient's MBD, and sufficient information to provide parents and patients with access to all the professionals involved in their care. It is important to have a standardized model that lists the documents and data summarized in Fig. 1. Some good examples for the preparation of these materials can be found in a series of websites that contain information and resources for the development of clinical transition programmes from paediatric to adult care (Table 5).

**Table 5:** Websites with information and resources for developing clinical transition programmes from paediatric to adult care

Florida: http://hctransitions.ichp.ufl.edu/index.php
Florida: www.floridahats.org
New York: http://healthytransitionsny.org
Washington, National HealthCare Transition Center: www.gottransition.org
Collaboration between Upstate Medical University, Golisano Children’s Hospital and NY State DD Planning Council: http://healthytransitionsny.org
Seattle: http://cshcn.org/teens
Kentucky: http://chfs.ky.gov/ccshcn/ccshcntransition.htm
Boston: http://www.childrenshospital.org/centers-and-services/programs/o-_-z/social-work/patient-resources/transitioning-to-adult-care
New England Consortium: https://newenglandconsortium.org/brochures/Transition-Toolkit-Complete.pdf
NICE (UK): https://www.nice.org.uk/Media/Default/About/NICE-Communities/Social-care/quick-guides/3312_SCIE_Planning%20for%20transition%20quick%20guide-09.pdf
UK: www.togetherforshortlives.org.uk
Melbourne: www.rch.org.au/transition/index.cfm?doc_id=8143
Toronto: www.hollandbloorview.ca/resourcecentre/transitions/adultservices.php

A key component of transitional care is the availability of appropriately trained personnel with skills and knowledge in the area. In fact, some studies suggest that the determinants of satisfaction among adolescents in transition include the availability of qualified staff, rather than the characteristics of the physical setting and problems during the process [9]. Unfortunately, however, professionals with knowledge of the transition process are not always available in many hospitals, and most respondents (Table 4) emphasized the need for training of paediatricians and adult specialists in the transition (95.3%), even in the form of regulated accreditation, although a lower degree of consensus was reached on this point (79.1%). It is understood that training should include both theoretical foundation training and care experience in functional transition units.

Training of a qualified team can not only help facilitate the patient’s transition, but can also serve to as a point of reference in the process, unifying referral, evaluation and follow-up criteria. It brings in not only specialist doctors and/or nurses, but also other figures such as psychologists, occupational therapists, social workers, physiotherapists and orthopaedic surgeons, and others whose participation is necessary in MBDs, and, in general, in any chronic disease that may involve some degree of disability/adaptation at such a critical stage as adolescence [53]. When planning a new transition unit, it may be useful to identify professionals who may be interested in transitional care and support them in their training, or find services located within the same hospital or referral hospital with already formed transition units [8].

#### Recommendations

The following materials should be included in the transition programme (Fig. 1):

- A letter of information for parents to be given at the time of diagnosis, or when the child is still under 12 years of age (Fig. 1, point 1), informing them about the future transition programme.
- A letter of information about the transition programme for the parents and another for the adolescent to be given when the child is between the ages of 12 and 18 (Fig. 1, points 2 and 3).
- The letter should include a summary of information about the disease for the patient and their family that includes general information about their disease, the general testing schedule and frequency of visits based on age, and access to medical societies and patient associations (Fig. 1, point 4).
- A clinical and progress report of the patient initiated by the paediatrician that will be transferred to the adult specialist (Fig. 1, point 5).
- A medical emergency card (Fig. 1, point 6) to be given to the patient.
- A contact card (Fig. 1, point 7) with the name and contact details of the case manager, paediatric and adult specialists, nurse manager and social worker.

### Barriers and evaluation of the effectiveness of the programme

For the development and implementation of a transition programme for patients with MBD, it is essential to identify any barriers that hinder this process and analyse how to address them [68].

The main barriers according to our survey are: (1) lack of resources (43.3%); (2) poor coordination and communication between paediatrics and adult services (19.3%); (3) some factors related to the low prevalence of MBD (12.9%); and (4) other barriers related to the adult specialty (11.2%) (Fig. 3).

To address the main barrier, human resources, administrative support, time dedicated to the transition, a suitable space, materials and adequate funding are required.

The poor coordination between paediatric and adult specialists may be explained by differences in patient management. The chronic paediatric patient receives supervised and monitored care. However, adult care involves multiple specialists that are usually poorly coordinated, generating “fragmented care”, which leads to a loss of confidence by the patient. A poorly coordinated transition results in confusion, lack of therapeutic compliance, emergency admissions and dependence on external caregivers [68]. A coordinated and gradual transition will build the adolescent’s confidence in the new team.

A central professional figure (nurse or case manager) capable of coordinating and acting as a link between specialists is important, guaranteeing continuity of care and prompt and effective information for the patient [64].

To address factors related to the low prevalence of MBD, training activities for professionals, patients and family members should be promoted, and specific clinical guidelines should be developed.

Regarding factors related to the adult specialty, the adult specialist often has limited knowledge of rare congenital MBDs, and their patients may arrive without a personalized plan or complete medical report from paediatrics. They may also lack skills for communicating with adolescents [3].

These difficulties are very similar to those reported in a study of internal medicine specialists on transition in general, the most relevant being the lack of training/knowledge of chronic childhood diseases [69]. We therefore believe that a transition programme should be established in coordination with the management teams, and that they should be provided with the necessary resources for implementation.

Once the transition programme has been created, it will be necessary to define quality and effectiveness indicators, and to gather this information periodically in order to carry out adequate quality control and continuous improvement [4, 8, 70].

The 2016 Cochrane review of the effectiveness of transition interventions recommended the evaluation of disease-specific patient outcomes as the primary outcome and, as secondary outcomes, the patient's readiness for the process, their satisfaction, treatment adherence, health-related quality of life, disease-related knowledge, self-advocacy skills, improvement in their safety, and healthcare resource use and costs [1, 68].

The effectiveness of the transition process should be evaluated by a dedicated questionnaire for patients and families in close collaboration with the coordinator. The satisfaction of the patient with the program as well as the trust in the whole team needs to be assessed by the coordinator and/or the specialised nurse or case manager. In addition, the program itself must put in place the mechanism to assess the compliance with the follow-up plan and the adherence to treatment until the transition is completed, a fact that must be highly individualized.

#### Recommendations

- Barriers in the hospital to implementing a transition programme should be identified, the most common being lack of resources, lack of coordination between paediatricians and adult physicians, lack of knowledge about rare diseases and lack of skills for communicating with the adolescent.
- Indicators or parameters should be designed to assess the effectiveness of the programme: complete documents that help the adult specialist to provide proper follow-up, patient support measures, identification of results that point to worsening of disease control, patient satisfaction survey and assessment of health-related quality of life.

## Conclusions

Despite growing interest in the transition of patients with chronic diseases from paediatric to adult care, few programmes are described in detail in the literature [64, 71–74]. The aim of the TEAM Project was to analyse the critical points during the transition process and to provide practical recommendations for creating a transition plan for children with MBD.

The limitations of the project were related to the number of doctors who eventually participated in the consensus, which was hampered by the emergence in 2020 of the SARS-CoV-2 pandemic, although the high degree of consensus obtained on the vast majority of the questions posed strengthens the findings. It would have been desirable to have the participation of other professionals involved in the transition process, such as nurses, psychologists and social workers, who were considered essential by physicians who responded to the survey. Likewise, obtaining information from families and patients would have complemented the opinion and needs of all those involved. The implication of the patient’s associations should also have been very relevant. The work carried out among the professionals responsible for this area could be an important initial step in setting up a comprehensive transition project that substantially takes into account the opinion of all stakeholders.

After creating the essential materials needed to establish a transition plan, the pertinent professionals must be informed of their availability and encouraged to use them. Facilitating the administrative process for parents and patients, giving priority to adolescent appointments during the transition period and avoiding duplication of visits and testing will contribute to the success of the programme and better patient care.

Twelve reviews assessing the effectiveness of transition programmes were identified: six focused on specific diseases such as diabetes [14, 15], palliative care [16], mental health [17], and spina bifida [18, 19], one review looked at chronic diseases [20], three reviews included studies in which patients had a range of medical needs or disabilities [21–23], and two reviews summarized evidence from qualitative studies on patients' perspectives [24, 25]. All reviews concluded that to reach valid conclusions, studies evaluating interventions require better designs that feature quantifiable quality and effectiveness indicators to demonstrate the extent of their benefits for patients and society, determined before and after starting the intervention programme.

The TEAM Project provides an overview of the transition of young patients with MBD to adult care in Spain and provides practical recommendations for implementing a transition model. However, before implementing this transitional approach, the endorsement of the patient's associations will be desirable.

### Supplementary Information

**Additional file 1.** Questionnaire for specialist consensus. Questionnaire used for specialist consensus.

## Data Availability

The datasets used and analysed during the current study are available from the corresponding author upon reasonable request and with permission from Kyowa Kirin Farmacéutica Data are located in controlled access data storage at E-C-BIO (https://www.ecbio.net).
