# Supplementary material for: Transition of patients with metabolic bone disease from paediatric to adult healthcare services: current situation and proposals for improvement
Source: Orphanet J Rare Dis. 2023 Aug 29;18:245. doi: 10.1186/s13023-023-02856-6 (PMC10463506; doi:10.1186/s13023-023-02856-6)
Supplement: Supplementary file 1 — Additional file 1. Questionnaire for specialist consensus. Questionnaire used for specialist consensus. [file 13023_2023_2856_MOESM1_ESM.doc]

| **INSTRUCTIONS** | |
| --- | --- |
| Thank you very much for your interest in participating in the TEAM PROJECT (Transition to Adult Care for Patients with Metabolic Bone Diseases). Please fill out the questionnaire below, which is divided into two parts:   1. **General information about you and your clinical experience** 2. **29-question questionnaire divided into four sections:**    1. RATIONALE FOR THE TRANSITION PROGRAMME AND START TIME    2. TRANSITION MODEL AND PLAN    3. INFORMATION IN THE TRANSITION PROGRAMME    4. TRANSITION DOCUMENTS AND TRAINING   You will find different types of questions: multiple-choice questions, questions with numerical answers and questions about your level of agreement with a statement. In the latter type of question, express your level of agreement on a 10-point scale ranging from 1 to 10, where 1 is "Disagree," 10 is "Completely agree," and the intermediate values are intermediate points between these two values.  **It is very important that you try to answer all the questions in the questionnaire. It will take no more than 15 minutes to complete.**  If you have any questions, you can contact us at: **proyectoTEAM@ecbio.net** | |
| **GENERAL INFORMATION ABOUT THE PARTICIPANT AND HIS/HER EXPERIENCE** | |
| 1. **Do you have any experience in the management of patients with metabolic bone disease?** | □ No  □ Yes |
| 1. **Approximately how many patients with metabolic bone disease have you treated? (Please put 0 if you have no experience)** |  |
| 1. **How many patients with X-linked hypophosphataemic rickets (XLH) have you treated? (Please put 0 if you have no experience)** |  |
| 1. **Age** |  |
| 1. **Gender** | □ Male  □ Female |
| 1. **Province** |  |
| 1. **Population predominantly treated** | □ Paediatrics  □ Adults  □ Both |
| 1. **Specialty** |  |
| 1. **Number of years of experience in the specialty (excluding residency)** |  |
| 1. **Scientific society or societies participating in the study, of which you are a member** | □ AECOM (Spanish Association for the Study of Inborn Errors of Metabolism)  □ AENP (Spanish Association of Paediatric Nephrology)  □ SEEN (Spanish Society of Endocrinology and Nutrition)  □ SEEP (Spanish Society of Paediatric Endocrinology)  □ SEIOMM (Spanish Society of Bone and Mineral Metabolism Research)  □ SEMI (Spanish Society of Internal Medicine)  □ SEN (Spanish Society of Nephrology)  □ SERPE (Spanish Society of Paediatric Rheumatology) |
| 1. **Type of hospital** | □ Public  □ Private  □ Mixed |
| 1. **Approximate number of patients you see per month in your clinic** | General patients: ……………  Patients with metabolic bone disease: …………… |
| 1. **Are you a part of or do you have experience in a healthcare transition unit, clinic or programme?** | □ No  □ Yes |

| **SECTION 1 - RATIONALE FOR THE TRANSITION PROGRAMME AND START TIME** | |
| --- | --- |
| 1. **Do you know what a transition unit, clinic or programme is?** | □ No  □ Yes |
| 1. **A care transition programme needs to be created for children with metabolic bone disease who transfer from paediatric to adult services** | Disagree ①②③④⑤⑥⑦⑧⑨⑩ Completely  agree |
| 1. **It is feasible to establish a transition programme for children with metabolic bone disease in your setting** | Disagree ①②③④⑤⑥⑦⑧⑨⑩ Completely  agree |
| 1. **List the THREE main barriers or difficulties to the establishment of a transition programme for children with metabolic bone disease in your setting** | 1.  2.  3. |
| 1. **At what age do you think it is best to start the transition programme in children with metabolic bone disease?** |  |
| 1. **It is appropriate to start the transition programme at 12 years old in patients with metabolic bone disease** | Disagree ①②③④⑤⑥⑦⑧⑨⑩ Completely  agree |
| 1. **It is feasible to start the transition programme at 12 years old in patients with metabolic bone disease** | Disagree ①②③④⑤⑥⑦⑧⑨⑩ Completely  agree |
| 1. **At what age do you think it is best to start the transition programme for children with X-linked hypophosphataemia (XLH)?** |  |
| 1. **In patients with XLH, which medical specialty should be the primary case manager?** | In paediatrics: …………………..  In adult patients: …………………… |
| 1. **Validated scales should be used to evaluate whether the adolescent is ready to start follow-up in adult specialties** | Disagree ①②③④⑤⑥⑦⑧⑨⑩ Completely  agree |
|  |  |
| **SECTION 2 - TRANSITION MODEL AND PLAN** | |
| 1. **Is there a transition model or plan in your hospital?** | □ No  □ Yes |
| 1. **Which transition model do you prefer in the care of patients with metabolic bone disease?** | □ Direct and complete transfer to the adult specialist when the patient turns 18  □ Gradual transfer to the adult specialist, with multidisciplinary paediatric-adult consultation during the transition  □ The patient is seen both in a paediatric clinic and an adult clinic, in different specialties  □ No transition, the patient remains with the paediatric specialist in the adult phase as well  □ Other type |
| 1. **The transition model you selected as your preferred model is feasible in your setting** | Disagree ①②③④⑤⑥⑦⑧⑨⑩ Completely  agree |
| 1. **There should be a transitional care unit/programme manager or case manager** | Disagree ①②③④⑤⑥⑦⑧⑨⑩ Completely  agree |
| 1. **It is feasible in your setting to appoint a transitional care unit/programme manager or case manager** | Disagree ①②③④⑤⑥⑦⑧⑨⑩ Completely  agree |
| 1. **Multidisciplinary working groups should be created for the management of adolescents with metabolic diseases** | Disagree ①②③④⑤⑥⑦⑧⑨⑩ Completely  agree |
| 1. **It is feasible in your setting to create multidisciplinary working groups for the management of adolescents with metabolic diseases** | Disagree ①②③④⑤⑥⑦⑧⑨⑩ Completely  agree |

| **SECTION 3 - INFORMATION IN THE TRANSITION PROGRAMME** | |
| --- | --- |
| 1. **Parents should be informed about the transition programme from the time the metabolic bone disease is diagnosed** | Disagree ①②③④⑤⑥⑦⑧⑨⑩ Completely  agree |
| **19. What is the minimum information that should be provided to the parents and the child during the transition programme?** | |
| - 1. **Letter of information for parents** | Disagree ①②③④⑤⑥⑦⑧⑨⑩ Completely  agree |
| - 1. **Letter of information for the adolescent** | Disagree ①②③④⑤⑥⑦⑧⑨⑩ Completely  agree |
| - 1. **Names and contact details of paediatric and adult specialists involved in patient care and contact details for nurse, social worker, and case manager** | Disagree ①②③④⑤⑥⑦⑧⑨⑩ Completely  agree |
| - 1. **Rough outline of visits and scheduled tests until the transition to adult care is complete** | Disagree ①②③④⑤⑥⑦⑧⑨⑩ Completely  agree |
| - 1. **Epidemiology of the disease, prevalence, incidence** | Disagree ①②③④⑤⑥⑦⑧⑨⑩ Completely  agree |
| - 1. **Diagnosis of the disease** | Disagree ①②③④⑤⑥⑦⑧⑨⑩ Completely  agree |
| - 1. **Prognosis of the disease, possible complications** | Disagree ①②③④⑤⑥⑦⑧⑨⑩ Completely  agree |
| - 1. **Frequency of disease control tests differentiating childhood, adolescence and adulthood** | Disagree ①②③④⑤⑥⑦⑧⑨⑩ Completely  agree |
| - 1. **Differences in treatment in childhood, adolescence and adulthood** | Disagree ①②③④⑤⑥⑦⑧⑨⑩ Completely  agree |
| - 1. **General health issues, diet, working life, smoking, alcohol, substances, fertility, contraception** | Disagree ①②③④⑤⑥⑦⑧⑨⑩ Completely  agree |
| - 1. **Patient associations and medical societies for information and advice** | Disagree ①②③④⑤⑥⑦⑧⑨⑩ Completely  agree |
| - 1. **Passport or card with information for management of the adolescent in case of emergency** | Disagree ①②③④⑤⑥⑦⑧⑨⑩ Completely  agree |
| 1. **Specific indicators of adequate metabolic disease control need to be identified** | Disagree ①②③④⑤⑥⑦⑧⑨⑩ Completely  agree |
| 1. **Planning for the time when the adolescent comes to the clinic alone, without their parents, is necessary** | Disagree ①②③④⑤⑥⑦⑧⑨⑩ Completely  agree |
| 1. **The adolescent’s degree of knowledge of the disease and its treatments should be assessed** | Disagree ①②③④⑤⑥⑦⑧⑨⑩ Completely  agree |
| 1. **The adolescent's satisfaction with the transition programme should be assessed** | Disagree ①②③④⑤⑥⑦⑧⑨⑩ Completely  agree |
| 1. **The role of the nurse in providing parents and children with information about the transition is important** | Disagree ①②③④⑤⑥⑦⑧⑨⑩ Completely  agree |
| 1. **The collaboration of the social worker in providing parents and children with information about the transition is important** | Disagree ①②③④⑤⑥⑦⑧⑨⑩ Completely  agree |

| **SECTION 4 - DOCUMENTS AND TRANSITION TRAINING** | |
| --- | --- |
| **26. What information do you think is essential to include in the transition report to another specialist in the care of the patient with metabolic bone disease?** | |
| - 1. **Demographic data** | Disagree ①②③④⑤⑥⑦⑧⑨⑩ Completely  agree |
| - 1. **Family history of disease** | Disagree ①②③④⑤⑥⑦⑧⑨⑩ Completely  agree |
| - 1. **Personal history of disease** | Disagree ①②③④⑤⑥⑦⑧⑨⑩ Completely  agree |
| - 1. **Date of diagnosis of metabolic bone disease** | Disagree ①②③④⑤⑥⑦⑧⑨⑩ Completely  agree |
| - 1. **History of previous treatments and efficacy obtained** | Disagree ①②③④⑤⑥⑦⑧⑨⑩ Completely  agree |
| - 1. **Complications of the disease and their treatment** | Disagree ①②③④⑤⑥⑦⑧⑨⑩ Completely  agree |
| - 1. **Current treatments** | Disagree ①②③④⑤⑥⑦⑧⑨⑩ Completely  agree |
| - 1. **Indicators for the control of metabolic bone disease** | Disagree ①②③④⑤⑥⑦⑧⑨⑩ Completely  agree |
| - 1. **Essential data to consider in the event of an emergency** | Disagree ①②③④⑤⑥⑦⑧⑨⑩ Completely  agree |
| 1. **Templates for transition documents may be useful** | Disagree ①②③④⑤⑥⑦⑧⑨⑩ Completely  agree |
| 1. **Paediatricians and specialists need to be trained in the transition to adult care programme** | Disagree ①②③④⑤⑥⑦⑧⑨⑩ Completely  agree |
| 1. **It is important to have accreditation in training in transition programmes** | Disagree ①②③④⑤⑥⑦⑧⑨⑩ Completely  agree |

| **THANK YOU FOR COMPLETING THE QUESTIONNAIRE** |
| --- |
